# Neural Acupuncture Unit: A New Concept for Interpreting Effects and Mechanisms of Acupuncture

**DOI:** 10.1155/2012/429412

**Published:** 2012-03-08

**Authors:** Zhang-Jin Zhang, Xiao-Min Wang, Grainne M. McAlonan

**Affiliations:** ^1^School of Chinese Medicine, LKS Faculty of Medicine, The University of Hong Kong, 10 Sassoon Road, Pokfulam, Hong Kong; ^2^National Institute of Nursing Research, National Institutes of Health, Bethesda, MD 20892, USA; ^3^Department of Forensic and Neurodevelopmental Science, Institute of Psychiatry, King's College London, London, UK

## Abstract

When an acupuncture needle is inserted into a designated point on the body and mechanical or electrical stimulation is delivered, various neural and neuroactive components are activated. The collection of the activated neural and neuroactive components distributed in the skin, muscle, and connective tissues surrounding the inserted needle is defined as a neural acupuncture unit (NAU). The traditionally defined acupoints represent an anatomical landmark system that indicates local sites where NAUs may contain relatively dense and concentrated neural and neuroactive components, upon which acupuncture stimulation would elicit a more efficient therapeutic response. The NAU-based local mechanisms of biochemical and biophysical reactions play an important role in acupuncture-induced analgesia. Different properties of NAUs are associated with different components of needling sensation. There exist several central pathways to convey NAU-induced acupuncture signals, Electroacupuncture (EA) frequency-specific neurochemical effects are related to different peripheral and central pathways transmitting afferent signals from different frequency of NAU stimulation. More widespread and intense neuroimaging responses of brain regions to acupuncture may be a consequence of more efficient NAU stimulation modes. The introduction of the conception of NAU provides a new theoretical approach to interpreting effects and mechanisms of acupuncture in modern biomedical knowledge framework.

## 1. Introduction

Modern acupuncture can be defined as a therapeutic technique in which sharp, thin needles are inserted into specific points on the body with mechanical, electrical, or other physical stimulation. The nomenclature and localization of most specific points, known as acupuncture points or acupoints, were established in traditional Chinese medicine (TCM) during about B.C. 400–A.D. 1740s. Over the past four decades, numerous clinical observations and studies have shown that acupuncture therapy possesses broad therapeutic benefits [1, 2]. A large body of experimental evidence obtained in animals and human subjects provides many insights into neural mechanisms of acupuncture effects, in particular acupuncture analgesia [3]. Today, this ancient healing technique is increasingly introduced into clinical practice, particularly for neuropsychiatric disorders [2]. Despite this, several fundamental issues remain unresolved in acupuncture research.

### 1.1. Metaphysical Concepts of Acupoint and Meridian in TCM

The doctrine of TCM was originally developed from elementary anatomical knowledge obtained in early days [4]. This is evidenced in numerous gross anatomical studies with measurement and a vast number of anatomical terms recorded in ancient TCM bibliographies. Ancient doctors had observed peripheral nerve trunks, branches, and plexus widely distributed in the superficial and deep tissues as well as on visceral organs, referring to as “meridians” and “collaterals” (*Jing* and *Luo* in Chinese). They believed that the meridians with the collaterals constitute an extensive network that communicates all parts of the body via the meridian energy (*Jing-Qi* in Chinese). The meridian energy can flow onto specific loci, termed “convergences” or “conjunctions” in ancient terms and “acupoints” today. The doctrine of TCM clearly states that acupoints are not the skin, muscles, connective tissues, or bones, but local sites where the meridian energy effuses onto the superficial tissues and infuses into the deep tissues and visceral organs [4].

Ancient doctors also had observed that pathological conditions occurred in the deep tissues and visceral organs can be manifested as pain or tender points on the body, called *A-Shi* points. The localizations and clinical indications for most meridian-based acupoints were initially developed from *A-Shi* points. Ancient doctors suggested that stagnation of the meridian energy is a determining factor in the pathogenesis of diseases. Needling, moxibustion, and other forms of stimulation on acupoints were considered to improve pathological conditions by unblocking the stagnation of the meridian energy and rearranging the balance of *Yin* and *Yang*, that is, homeostasis.

It would seem that the metaphysical concepts of acupoint and meridian represent an entity with particular anatomical and physiological neural profiles. Nevertheless, how to precisely elucidate the metaphysical concepts of acupoint and meridian in the framework of modern biomedical knowledge has been a key issue in acupuncture research.

### 1.2. “Specific” and “Nonspecific” Properties of Acupoints

As acupoints are deemed “specific” points in the doctrine of TCM, many efforts have been made to identify their “specific” properties. Potential differences between the traditionally defined acupoints and so-called “nonacupoints” have been examined at anatomical, histological, biochemical, and electrophysiological levels in both animals and human subjects [4]. Although early studies indicate that most acupoints are located on or adjacent to peripheral nerve trunks or branches, and the meridians correspond with trajectories of relevant peripheral nerves [4], there is no convincing evidence to support the existence of novel or special structures beneath acupoints. However, histological studies indeed have revealed a relatively dense and concentrated distribution of certain neural and neuroactive components beneath many acupoints commonly used in clinical practice compared to adjacent areas [4]. Electrophysiological studies also have shown that the skin along with acupoints and meridians may possess distinct electrical properties which are closely associated with the activity of local neural and neuroactive components [5]. These results suggest the relativity of the “specific” and “nonspecific” properties of acupoints.

The definition and identification of the pattern of “specific” and “nonspecific” neural and neuroactive components in the response to acupuncture stimulation would help us better understand the essential mechanisms of acupuncture and develop more efficient acupuncture stimulation modes. However, the metaphysical concept of acupoint and meridian itself cannot provide sufficient information for defining and identifying the response pattern. Interactions between neural and neuroactive components as well as the relationship with the local and central response to acupuncture stimulation are also not well elucidated. Thus, it was necessary to introduce an alternative concept that substantially differentiates from the metaphysical concept of acupoint. Such concept would provide a more accurate term and a new theoretical approach to interpreting effects and mechanisms of acupuncture.

## 2. The Definition of Neural Acupuncture Unit (NAU) and Its Differentiation from Acupoint

Insertion into the skin with filiform needles is the most commonly used form of acupuncture stimulation in clinical practice. When a filiform needle is inserted into a designated point on the body and mechanical (manual manipulation) or electrical stimulation is delivered, a variety of neural and neuroactive components are activated. A collection of the activated neural and neuroactive components distributed in the skin, muscle, and connective tissues surrounding the inserted needle is defined as a neural acupuncture unit (NAU). Here, the designated points include not only the traditionally defined acupoints, which are often called as meridian-based acupoints or acupoints in short, but also *A-Shi* points and control points (sometimes called non-acupoints or placebo points) as specifically designated in acupuncture research.

**Figure 1:**
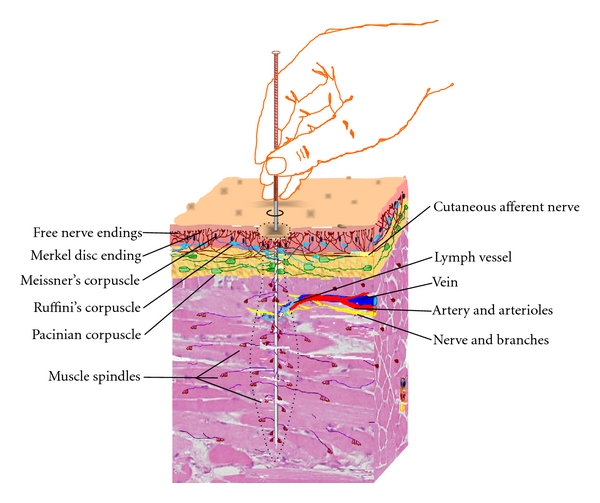
A representative muscle-spindle-rich NAU in the response to manual twists of acupuncture stimulation. The NAU with the related neural and neuroactive components is illustrated as the dotted line-defined vase-like pattern, which is principally determined by twist-produced different distant effects on mechanoreceptors located in cutaneous and muscle tissues (see Section 5.1).

Apparently, NAU is a hypothetical concept that represents local neural and neuroactive components in the physiological, biochemical, and therapeutic response to needling stimulation, rather than localization of the stimulation. On the other hand, viewed from ancient and modern anatomical perspective [4], the traditionally defined acupoints could be defined as an anatomical landmark system that indicates local sites where NAUs may contain relatively dense and concentrated neural and neuroactive components, upon which acupuncture stimulation would elicit a more efficient therapeutic response compared to nonacupoints. In theory, there are innumerable NAUs existing in the body as acupuncture procedure can be performed in different directions at the same point and on most areas of the body, including myriad *A-Shi* points and 361 WHO-defined standard acupoints [6]. The pattern of NAUs varies, mainly depending upon designated points and acupuncture stimulation mode as well as needling direction and depth. A hypothetical NAU is illustrated in Figure 1.

## 3. Major Neural and Neuroactive Components of NAUs

### 3.1. Neural Components

Early studies in animals and human autopsies revealed that most acupoints contained abundant free nerve endings, encapsulated cutaneous receptors (Merkel, Meissner, Ruffini, and Pacinian corpuscles), sarcous sensory receptors (muscle spindles and tendon organs), and their afferent fibers [4]. Somatic efferent fibers innervating muscles, small nerve bundles, and plexus were also observed in acupoint tissues, but no novel structures were found beneath acupoints. Many acupoints examined had relatively dense neural components, particularly nerves fibers, with a ratio of nearly 1.4 : 1 compared to non-acupoint areas [7–9]. The ratio of local myelinated to nonmyelinated fibers was found to be nearly 4-fold higher than surrounding areas in human Zu-San-Li (ST36) [9, 10]. A similar phenomenon was also recorded in rats, showing that sarcous sensory receptors and their afferent fibers are concentrated at acupoints located on thick muscles [11].

Another important neural component of NAUs is dense and fine autonomic nerve fibers [12]. A close approximation of autonomic fiber varicosities and somatic afferent fiber terminals is often observed in rabbit acupoint areas [12]. Most autonomic nerves are noradrenaline- (NA-) containing sympathetic fibers, and an interaction between somatic and autonomic neural components may serve to modulate local and afferent signals in NAUs (see below).

### 3.2. Neuroactive Components and Related Mediators

**Figure 2:**
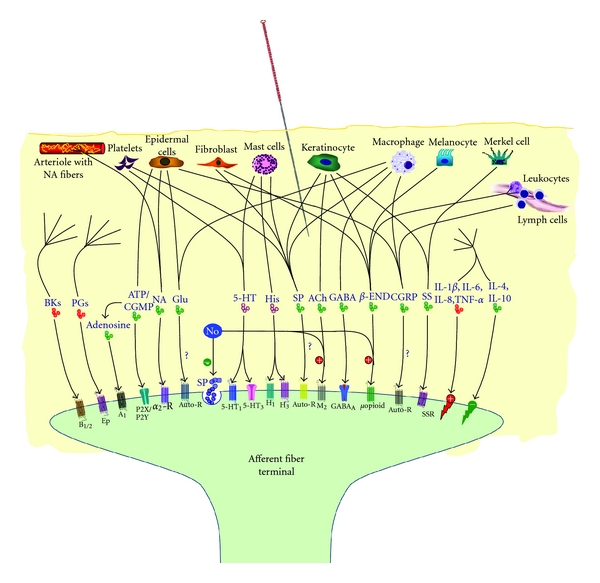
Schematic illustration of major nonneuronal neuroactive mediators and their corresponding receptors involved in the modulation of NAU afferent impulses. Molecules in red, green, and violet color represent stimulatory, inhibitory, and both effects on afferent fiber excitability, respectively. Autoreceptors to be identified are indicated with question symbols (?). A_1_, adenosine A_1_ receptor; ACh, acetylcholine; Auto-R, autoreceptor; B_1/2_, bradykinin receptors 1 and 2; BK, bradykinin; CGRP, calcitonin-gene-related peptide; *β*-END, *β*-endorphin; EP, prostaglandin E receptor; GABA, *γ*-aminobutyric acid; Glu, glutamate; H_1_/H_2_, histamine H_1_/H_2_ receptors; His, histamine; 5-HT, 5-hydroxytryptamine; IL, interleukin; M_2_, muscarinic M_2_ receptor; NA, noradrenaline; NO, nitric oxide; PG, prostaglandins; P2X/P2Y, purinergic receptors P2X and P2Y; *α*_2_-R, *α*_2_ adrenoceptor; SP, substance P; SS, somatostatin; SSR, somatostatin receptor; TNF-*α*, tumor necrosis factor-*α*.

Broadly speaking, neuroactive components of NAUs can be defined as nonneuronal tissues and cells that release various mediators capable of modulating afferent fiber transmission of NAUs. The most apparent neuroactive components are mast cells, sympathetic nerve-rich blood vessels, and small lymphatic vessels [4]. In addition to mast cells that release many neuroactive mediators, including histamine, substance P (SP), and other immune factors via a degranulation mechanism in response to acupuncture stimulation [13–15], other non-neuronal cells, including macrophages, fibroblasts, lymphocytes, platelets, and keratinocytes are also involved in the modulation of local and afferent signals of NAUs. These cells release various transmitters, modulators, inflammatory and immune factors, which directly or indirectly act at corresponding receptors on the surface of peripheral afferent fibers (see below).  Table 1 summarizes major non-neuronal cell-released neuroactive mediators and their corresponding receptors. Based on their effects on afferent fiber excitability of NAUs, the mediators can be classified as inhibitory and stimulatory. The inhibitory mediators mainly include acetylcholine, noradrenaline (NA), *γ*-aminobutyric acid (GABA), *β*-endorphin, SP, somatostatin, nitric oxide (NO), ATP/cGMP, and adenosine, which suppress afferent fiber excitability of NAUs. Most cytokines, prostaglandins, bradykinin, and other proinflammatory factors are stimulatory mediators that directly or indirectly enhance afferent fiber excitability of NAUs. Serotonin (5-HT) and histamine can exert either inhibitory or stimulatory effects, depending upon which receptors they act on (Figure 2).

## 4. Biochemical Reactions of NAUs

When an acupuncture needle is inserted into a designated point and repetitively manipulated in different directions, it is assumed to cause local tissue injury and biochemical reactions, with the release of various inflammatory and immune mediators in NAUs. Nevertheless, unlike most other forms of tissue injury, acupuncture-induced tissue injury may represent a “positive” biochemical process resulting in therapeutic responses at local and systemic levels. This is considered due to a robust axon reflex and modulation of afferent fiber transmission in NAUs.

### 4.1. Acupuncture-Induced Robust Axon Reflex in NAUs

**Figure 3:**
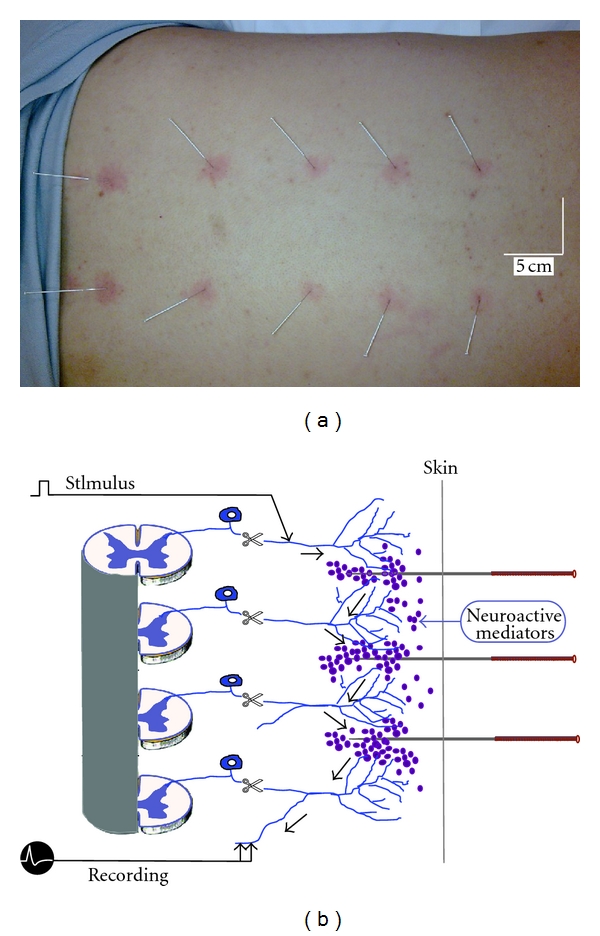
Acupuncture-induced robust axon reflex (a) and its involvement in the propagated sensation along meridians (PSM) (b). In (a), hyperemia (flare) was induced by acupuncture needling in acupoint areas of Bladder Meridian Foot Taiyang in the back. (b) illustrates putative communication between adjacent branches of nerves from different spinal segments via neuroactive mediators released by acupuncture stimulation from neural and non-neuronal tissues. (b) was reproduced based on the work done by Professor Zhao's research group with his generous permission (also see [20, 21, 224]).

The axon reflex is a response to peripheral tissue injury, which produces an impulse that moves from one nerve branch to other branches in close contact with nonneural tissues, mainly blood vessels, sweat glands, and mast cells [16]. This results in vasodilatation and release of vascular and neuroactive mediators from immune cells leaking from dilated vessels [17]. It is well documented that the axon reflex plays a central role in immune-nerve crosstalk, especially in neurogenic inflammation [17]. Clinical observations have demonstrated that acupuncture-induced axon reflex is strongly apparent in acupoint areas, particularly in the back and abdominal acupoints. It is characterized by a hyperemia (flare) that rapidly (generally within 2–5 min) spreads beyond needling points of the skin with a diameter of 1–3 cm (Figure 3(a)). Moreover, the acupuncture-evoked flare sometimes can spread along a meridian across several dermatomes supplied by nerves from totally different spinal segments, becoming a red line accompanied by the propagation of the needling sensation. This phenomenon is called the propagated sensation along meridians (PSM), which occurs in 0.3% of the healthy population [18].

The robust axon reflex of NAUs and PSM observed during acupuncture stimulation may be closely related to dense sympathetic nerve-rich arterioles, lymphatic vessels, and mast cells as well as concentrated primary afferent fibers in NAUs [4, 19]. The acupuncture-induced robust axon reflex is more likely due to transient vasodilatation and temporary neural communication between adjacent branches of nerves from different spinal segments via vascular and neural mediators released from neural and nonneuronal tissues (Figure 3(b)) [15, 20, 21]. Apparently, the robust axon reflex plays an important role in the production of local and afferent signals in NAUs [19].

### 4.2. Local Modulation of NAU Afferent Impulses by Neuroactive Mediators

In response to acupuncture-caused tissue injury, mast cells, platelets, and other immune cells migrate to make close contact with afferent nerve terminals in NAUs. The injured and migrated cells consequently release various neuroactive mediators, which infiltrate the tissues and act at corresponding receptors on the surface of afferent nerve terminals in NAUs via the axon reflex [17]. Meanwhile, tissue injury results in plastic changes in peripheral primary afferents, which develop synapse-like contacts with postganglionic sympathetic nerve varicosities, where NA release acts on *α*-adrenoceptors on afferent nerve terminals of NAUs [22, 23].

Although acupuncture-caused tissue injury could induce the release of both inhibitory and stimulatory mediators from non-neuronal cells in NAUs via the robust axon reflex, as shown in Table 1, inhibitory mediators released may predominate over stimulatory mediators. Several lines of evidence indicate that the predominant effect of acupuncture is to enhance the activity of inhibitory mediators under pain conditions. First, compared to most other tissue injuries, acupuncture-caused tissue injury is minimal. This may mean that the release of proinflammatory factors, most of which enhance NAU afferent fiber excitability, is limited. On the other hand, a large number of studies have shown that acupuncture significantly elevates the concentrations of many non-neuronal cell-released neuroactive mediators in local tissues at acupoints, especially including NA [24–26], *β*-endorphin [27, 28], somatostatin [29], and acetylcholine (ACh) [30], all of which suppress afferent fiber excitability of NAUs. Second, it is generally accepted that acupuncture is not only a noxious stimulus but is also mechanical and can be electrical (see below). Many inhibitory mediators, such as ATP and its metabolite adenosine, are released in response to mechanical and electrical stimulation [31]. Acupuncture stimulation has been found to robustly increase the extracellular concentrations of ATP and adenosine in mice's acupoint tissues, while analgesic effects were elicited [31].

Lastly, it is well known that a majority of peripheral small-diameter afferent fibers of the spinal and cranial nerves use the excitatory amino acid glutamate (Glu) as a transmitter [32, 33]. Most Glu-containing afferent fibers cocontain one or more peptides. Substance P (SP) and calcitonin gene-related peptide (CGRP) are the most common peptides that are colocalized in a majority of afferent fibers [34]. Many non-neuronal cells also synthesize and release Glu, SP, and CGRP as shown in Table 1. It has been proposed that peripheral afferent fibers bear autoreceptors for Glu [35, 36], SP [37, 38], and CGRP [39]. A number of studies in rats and humans have shown that electroacupuncture increased the quantity of CGRP [40–42] and SP [42–44] in peripheral tissues and in blood circulation, although the effects on local non-neuronal glutamate and other excitatory transmitters are unclear. The increased mediators in local tissues are believed to at least partly come from non-neuronal cells, accounting for nearly 50% of total levels of SP in rodent peripheral tissues [45, 46]. The elevation of non-neuronal mediators activates the negative feedback mechanism by acting at corresponding autoreceptors and, in turn, suppresses afferent fiber excitability of NAUs. 

Taken together, it can be assumed that, in addition to central mechanisms, NAU-based local mechanisms play an equally important role in acupuncture analgesia, via which afferent noxious signals from sites distal to needling points are blocked mostly by enhancing the activity of inhibitory mediators and activating the autoreceptor-based negative feedback in NAUs. There have been many studies proving the NAU-based local mechanism of acupuncture analgesia. A recent study found that, while manual acupuncture on rat Zu-San-Li (ST36) produced pronounced analgesic effects, it also enhanced the degranulation of mast cells in local acupoint tissues; however, the analgesic effects were completely abolished by local injection of disodium chromoglycate, an inhibitor of mast cell degranulation, indicating the involvement of mast cell-released mediators in acupuncture analgesia [15]. Local injection of naloxone, an opioid receptor antagonist, an antibody against *β*-endorphin, or corticotropin-releasing factor antagonist also eliminated analgesic potency of electroacupuncture (EA) in animal models of acute and chronic inflammatory pain [47, 48]. Likewise, subcutaneous acupoint injection of neostigmine, a cholinesterase inhibitor, significantly enhanced pain-relieving effects of EA in rats [30]. These studies suggest that the local *β*-endorphin and ACh play a key role in the local mechanism of analgesic effects of acupuncture. 

Most recently, it was found that, while acupuncture on mice's Zu-San-Li (ST36) significantly reduced chronic pain in the ipsilateral paw and increased the extracellular concentrations of ATP and adenosine in acupoint tissues, the local application of 2-chloro-N(6)-cyclopentyladenosine (CCPA), an adenosine A_1_ receptor agonist, replicated the analgesic effect of acupuncture. The local inhibition of enzymes involved in adenosine degradation also potentiated the acupuncture-elicited increase in adenosine, as well as its antinociceptive effect [31]. These data strongly suggest that acupuncture-released ATP and its metabolite adenosine in local acupoint tissues block pain impulses from sites distal to needling point.

### 4.3. The Relationship between NAUs and Electrodermal Properties of Acupoints

It is well documented that many immune mediators, in particular, local tissue-released NA, nitric oxide (NO), tumor-related factors, and mast cell-released histamine and 5-HT [15, 24, 49–52], are heavily involved in the determination of electrical properties of acupoints and meridians, namely, higher conductance, lower impedance, and higher capacitance compared to adjacent tissues [5]. Electrodermal measures have been shown to be significantly associated with clinical outcomes of acupuncture treatments in patients with chronic pelvic pain [53]. Acupuncture stimulation was found to change the human skin sympathetic nerve activity [54]. Normalization of skin electrical conductance at related acupoints has also been linked to therapeutic responses to acupuncture in subjects with heart stress [55], obesity [56], and acute joint injury [57]. These observations suggest that normalization of electrodermal properties at acupoints is perhaps associated with the modulation of neuroactive mediators in acupuncture stimulation. Whether electrodermal measures of acupoints could serve as a reliable and valid approach in detecting biochemical properties of NAUs deserves further investigation.

## 5. Biophysical Reactions of NAUs

**Figure 4:**
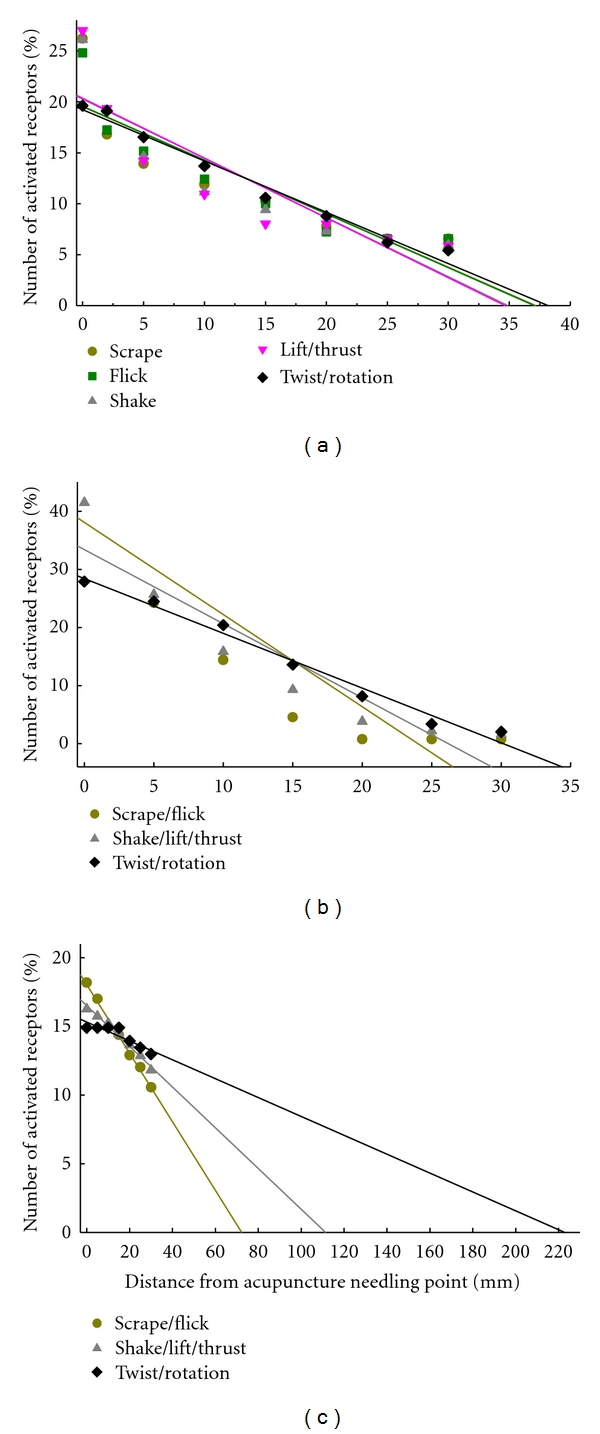
The distant effects of different manual techniques of acupuncture in activating cutaneous superficial mechanoreceptors (a), deep pressure-detected receptors (b), and muscle stretch receptors (c) located in the posterior aspect of the leg of Bladder Meridian Foot Taiyang in rabbits. Percent of the number of the activated receptors in each defined area surrounding needling point was calculated from the total number of the activated receptors. The plots were produced based on the data reported in [58, 59].

**Figure 5:**
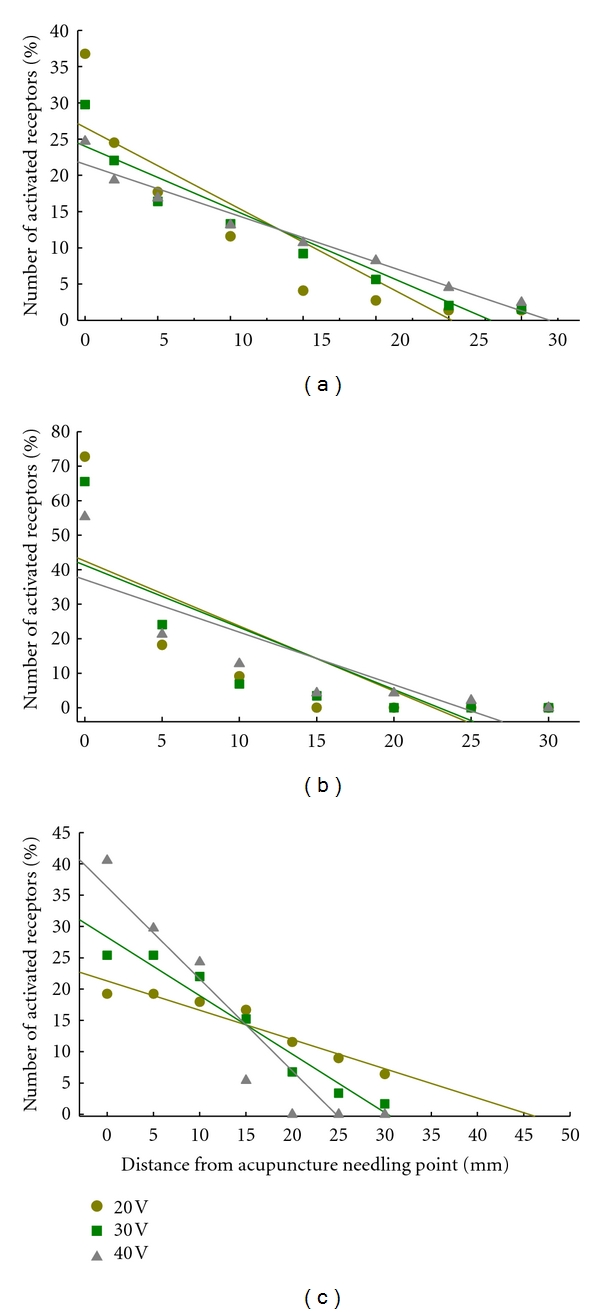
The distant effects of different intensities of electroacupuncture (EA) in activating cutaneous superficial mechanoreceptors (a), deep pressure-detected receptors (b), and muscle stretch receptors (c) located in rabbit triceps surae muscle areas of Bladder Meridian Foot Taiyang. Percent of the number of the activated receptors in each defined area surrounding needle point was calculated from the total number of the activated receptors. The plots were produced based on the data reported in [60, 61].

As mentioned above, acupuncture is not only a noxious stimulus but can include mechanical and electrical stimulation. Thus, apart from biochemical reactions, acupuncture also elicits biophysical reactions in NAUs. Early studies in rabbits have examined the responses of different types of NAU mechanoreceptors to different manual techniques and intensities of electrical stimulation [58–62]. The studies have revealed that the activation of the mechanoreceptors is not necessarily limited surrounding needling point but also can spread to a distance from needling point and this is referred to as “distant effect.” Moreover, there are high negative linear correlations between the number of the activated receptors and distance from needling point (see Figures 4 and 5). The distant effect is, therefore, a most important component of the biophysical reactions of NAUs.

### 5.1. Manual Acupuncture- (MA-) Induced Distant Effects

In acupuncture practice, manual manipulation is often performed on the inserted needles to enhance needling sensation and therapeutic responses. The most commonly used manual techniques include lift, thrust, twist, rotation, shake, scrape, and flick. Gentle and repetitive manipulation onto the inserted needle would be expected to produce mechanical pressure and tissue distortion that activate NAU mechanoreceptors located in the skin, muscle, and tendon tissues [58–63]. This mechanical effect has been well confirmed in recent studies of both mice and human subjects [64, 65].

It is well documented that the distant effect is mainly achieved by shear force- and stress-induced tissue displacements during manual manipulation [63–65]. All types of manual techniques tested have yielded greater distant effects on sarcous stretch receptors than cutaneous mechanoreceptors; twist/rotation has the greatest distant effects on the cutaneous superficial and deep receptors as well as sarcous stretch receptors compared to other techniques in rabbits (Figure 4) [58, 59]. The order of the distant effects of twist/rotation is sarcous stretch receptors, cutaneous superficial mechanoreceptors, and deep pressure-detected receptors. Based on this order, the twist-associated muscle-spindle-rich NAUs can be proposed to be a vase-like pattern as illustrated in Figure 1.

All types of manual techniques tested can activate A*α*, *β*, and *δ* fibers of NAUs. Twist/rotation additionally excites C-fibers on most occasions, whereas other types of manual techniques seldom do so [62, 66].

### 5.2. Electroacupuncture- (EA-) Induced Distant Effects

Electroacupuncture (EA) stimulation produces the distant effects in exciting cutaneous mechanoreceptors and sarcous stretch receptors of NAUs, with a range of nearly 25–45 mm from needling point. The EA intensity-dependent distant effect was observed on only the cutaneous superficial receptors, but not the cutaneous deep receptors and sarcous stretch receptors in rabbits (Figure 5) [60, 61].

Collectively, while most nociceptors are innervated by thin myelinated A*δ* and C fibers, most somatic mechanoreceptors are innervated by A*β* fibers. Therefore, the activation of mechanoreceptors and their A*β* afferent fibers appears to play a dominant role in the biophysical reactions of NAUs, particularly in muscle-spindle-rich NAUs.

## 6. NAU Classification and Its Differential Effects

### 6.1. Classification of NAUs

It is well documented that somatosensory receptors and their afferent fibers play the central role in the production of NAU afferent impulses [3]. Based on the predominance of somatosensory receptors, NAUs can be roughly classified into the three types: muscle-spindle-rich NAUs, cutaneous-receptor-rich NAUs, and tendon-organ-rich NAUs.  Table 2 summarizes the definition, characteristics, and related acupoints of the three types of NAUs.

### 6.2. Differential Properties of Afferent Impulses Produced in Different Types of NAUs

It is generally accepted that NAU afferent impulses are initially produced through biochemical and biophysical reactions and transmitted dominantly by thin fibers (A*δ* and C fibers) and thick fibers (A*β* fibers), respectively. The impulses represent therapeutic information that mainly consists of both “positive” tissue injury-induced and mechanoreceptor-activated signals. However, the predominant components may vary, largely depending upon different types of NAUs. For most muscle-spindle- and tendon-organ-rich NAUs, the stretch receptor-activated signals dominate NAU afferent impulses. This assertion is supported by an early study on acupuncture analgesia in healthy volunteers, revealing that increased pain threshold produced by manual acupuncture at He-Gu (LI4) was completely reversed by blockade of deep nerve branches innervating muscle fibers, but not cutaneous nerve branches [67]. Similar phenomena were also observed in acupuncture modulation of visceral functions in anesthetized rats, revealing that arterial blood pressure and heart rate were significantly reduced by manual acupuncture on acupoints with the muscles alone, but not the skin alone [68, 69]. Likewise, bidirectional rotation of a needle deeply inserted into a muscle-spindle-rich NAU beneath the human acupoint Shou-San-Li (LI10) produced greater needle sensation intensities compared to superficial needle insertion with mock deep penetration and bidirectional rotation [70].

In contrast, for most cutaneous-receptor-rich NAUs, for example, Ren-Zhong (GV26) and Shi-Xuan (EX-UE11), which are often used as consciousness-awakening, spirit-quieting, and mind-stabilizing acupoints for acute and severe neuropsychiatric conditions, such as summer stroke, shock, coma, acute fever-caused convulsion, trance, manic episode, and severe depression, the treatment effects are closely associated with patients' strong feeling of sharp pain evoked by pricking on the acupoints [71]. It appears that “positive” tissue injury-induced signals transmitted by small-diameter afferent fibers (mainly A*δ* and C fibers) may dominate afferent impulses from cutaneous-receptor-rich NAUs.

### 6.3. The Relationship between NAU Properties and Components of Needling Sensation

A large body of empirical and experimental evidence confirms that acupuncture stimulation with and without accompanying needling sensation (*De-Qi* in Chinese) leads to notable differences in neuroimaging [72, 73], electroencephalogram [74], and clinical outcomes [75]. Needling sensation is, therefore, suggested to be a predictor for acupuncture analgesia [76]. Although the perception of needling sensation may vary in individuals and with manual techniques, this distinct sensation is generally characterized by soreness, numbness, heaviness, distension, and aching in the deep tissues surrounding the inserted needle [76], and often accompanies increased blood flow with a feeling of warmth at acupoint areas [77, 78]. The sensation also can be transmitted to the acupuncturist's fingers, which feel increased resistance to further movement of the inserted needle [79]. Thus, the needling sensation is not a single, but a compound sensation that is generated from the activation of various sensory receptors and their afferent fibers in NAUs, in particular, small fiber-innervated nociceptors and myelinated fiber-innervated mechanoreceptors, which, respectively, produce afferent impulses via biochemical and biophysical mechanisms of NAUs as described.

It is also well demonstrated that numbness, heaviness, and distension during needling are closely associated with the activation of myelinated A*β* and A*δ* afferents in deep issues of acupoints, whereas aching and soreness are highly correlated with stimulation of small myelinated A*δ* and unmyelinated C fibers [10, 80–82]. Clinical practice also suggests that numbness, heaviness, and distension are more often elicited when manual manipulation is performed in muscle-spindle- and tendon-organ-rich NAUs, whereas the sensation evoked in cutaneous-receptor-rich NAUs is dominated by aching and soreness. The putative relationship between NAU properties and components of needling sensation is summarized in Table 3.

## 7. Multiple Central Neural Pathways Conveying NAU Afferent Impulses

**Figure 6:**
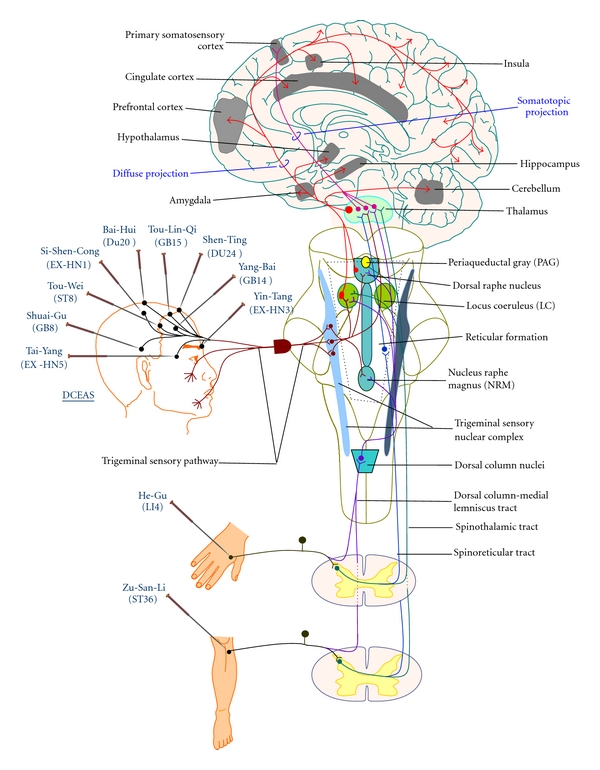
Schematic illustration of multiple central neural pathways transmitting NAU afferent impulses from different parts of the body. The brain areas commonly observed in neuroimaging response to acupuncture stimulation are indicated with gray shadow. DCEAS: dense cranial electroacupuncture stimulation.

As described above, acupuncture-evoked afferent impulses in most NAUs are mainly constituted by “positive” tissue injury-induced and mechanoreceptor-activated components. Neuroanatomically, there exist separate central pathways processing NAU afferent impulses from different components and from different parts of the body. Several spinal-supraspinal pathways responsible for acupuncture analgesia have been identified [3, 83]. The trigeminal sensory pathway is involved in the transmission of NAU afferent impulses from the trigeminal territory [84]. Via these pathways, most NAU afferent signals are carried up to the brainstem, where the signals are relayed to other subcortical and cortical areas via direct projections and collateral branch connections. Major central neural pathways processing NAU afferent impulses are illustrated in Figure 6.

### 7.1. The Spinal-Supraspinal Pathways

The spinal-supraspinal pathways responsible for transmitting NAU afferent impulses from the territory innervated by the spinal nerves mainly comprise the spinothalamic tract, the spinoreticular tract, and the dorsal column-medial lemniscus tract. Most peripheral small afferent fibers bearing “positive” noxious signals from NAUs in the limbs, the trunk, and the neck terminate in the superficial layers of the spinal dorsal horn, where the signals are relayed and carried up by the contralateral spinothalamic tract to supraspinal levels [83]. Most peripheral thick myelinated afferent fibers bearing NAU mechanoreceptor-activated signals in the spinal nerve territory separately enter the ipsilateral dorsal column-medial lemniscus tract and emerge into the contralateral spinothalamic tract. NAU impulses conveyed by the spinothalamic tract and the dorsal column are further relayed in the brainstem and the thalamus and ultimately sent to the somatosensory cortex in a somatotopic fashion [83]. Parallel to the somatotopic pathways, the spinoreticular tract receives NAU impulses largely via collateral connections with the somatotopic pathways at the spinal and supraspinal levels and diffusely projects to subcortical and cortical areas [83].

It is well documented that the spinothalamic and spinoreticular tracts are the two key ascending pathways, which convey NAU “positive” tissue injury-evoked signals and activate the descending noxious inhibitory system [3, 83]. The latter mainly consists of the periaqueductal gray (PAG) nucleus raphe magnus (NRM) spinal pathway and the locus coeruleus (LC)-spinal pathway. These send inhibitory information to the spinal dorsal horn and block noxious signal inputs from the periphery [3, 83, 85]. In addition to receiving signals from the ascending pathways, the descending inhibitory system also receives wide afferent modulation from supra-brainstem regions, including the hypothalamus, amygdala, prefrontal, anterior cingulated, and orbital cortex [83, 86]. 

As NAU mechanoreceptor-activated signals are believed to be the dominant components of afferent impulses in the majority of NAUs and the dorsal column-medial lemniscus tract receives multiple sources of sensory information, including cutaneous pain and visceral sensations in addition to fine touch and proprioception [87], it is thought that the dorsal column may play an equally important role in the processing of NAU afferent impulses. It has been shown that low-frequency EA stimulation (3 pulses/s) at acupoints in the rat hindlimbs increased neuronal nitric oxide synthase (NOs) expression in the gracile nucleus, an important relay of the rat dorsal column-medial lemniscus tract [88]. Repeated low-frequency EA stimulation of Guan-Yuan (CV4) in ovariectomized rats also enhanced the activity of neuronal cells in the cuneate nucleus, another important relay of the dorsal column [89]. However, cardiovascular responses induced by biphase-pulse electrical stimuli (3–30 pulses/s) at Zu-San-Li (ST36) were attenuated by blockade of neuronal conduction in the rat gracile nucleus [90]. These data suggest the involvement of the dorsal column-medial lemniscus tract in the modulation of acupuncture effects, particularly in regulating visceral functions [91]. This is consistent with the central actions of transcutaneous electrical nerve stimulation (TENS), of which the activation of the dorsal column pathway is believed to be the principal mechanism [92].

### 7.2. The Trigeminal Sensory Pathway

Sensory information from the face and the forehead are principally conveyed inward by the trigeminal nerve to the brainstem trigeminal sensory nuclear complex [84]. While NAU afferent impulses in the trigeminal territory are transmitted to the somatosensory cortex, neuroanatomical studies suggest that, compared with the spinal-supraspinal pathways, the trigeminal sensory pathway has much closer connections with the brainstem reticular formation, particularly with the dorsal raphe nucleus (DRN) [93, 94] and the locus coeruleus (LC) [95–98]. The latter two brain structures are the major resources of 5-HT and noradrenergic neuronal bodies, respectively, and play a pivotal role in the regulation of sensation, emotion, sleep, cognition, and visceral information processing [99, 100]. Furthermore, both low- and high-frequency EA stimulation has been reported to significantly increase the expression of 5-HT in the rat DRN [101–103] and suppress stress-induced increase in *c*-fos and tyrosine hydroxylase expression in LC [85, 104, 105].

The closer connections between the trigeminal sensory pathway and the brainstem reticular formation, in particular serotonergic and catecholaminergic neuronal systems, provide an important neuroanatomical rationale for the utilization of an efficient stimulation modality called dense cranial electroacupuncture stimulation (DCEAS). In this technique, electrical stimulation of dense acupoints located on the forehead is expected to produce a robust therapeutic response (Figure 6). Several pilot studies have confirmed the efficacy of DCEAS in the treatment of headache [106], refractory obsessive-compulsive disorder (OCD) [107], major depression [108], poststroke depression [109], and vascular dementia [110]. Most recently, we further demonstrated the effectiveness of DCEAS as an additional therapy in enhancing the antidepressant response to fluoxetine, a selective 5-HT reuptake inhibitor in the early phase of the treatment of major depression [111].

## 8. NAU-Associated Central Effects of Acupuncture

### 8.1. The Widespread Brain Regional Response: Neuroimaging Evidence

Over the past two decades, thanks to technological advantages in the spatiotemporal mapping of regional brain functions, neuroimaging approaches, such as functional magnetic resonance imaging (fMRI) and positron emission topography (PET), have been widely introduced into acupuncture research. The initial aim of these studies is to identify brain regional and functional correlates of acupoints and acupuncture stimulation modes [112].

Although several studies have shown that acupuncture stimulation on acupoints traditionally used for the treatment of vision and hearing disorders are correlated with the activation of corresponding visual and auditory cortex, respectively [113–115], methodological heterogeneity and poor replication have raised the criticism that the response is caused by methodological flaws, rather than a direct result of specific effects of acupuncture [116, 117]. In fact, apart from the somatotopic representation of acupoints in the primary somatosensory cortex, no well-defined correlations between distinct brain regional response patterns and a given acupoint or acupuncture stimulation mode have been identified. Instead, the vast majority of studies have consistently revealed that manual and electrical stimulation of single acupoints, represented by Zu-San-Li (ST36), He-Gu (LI4), Nei-Guan (PC6), Yang-Ling-Quan (GB34), Tai-Chong (LV3), Wai-Guan (TE5), and Guang-Ming (GB37), produces widespread modulation of cortical, limbic, subcortical, and brainstem areas through activation or deactivation in healthy volunteers [112]. Moreover, several brain regions display response patterns unrelated to acupoints and stimulation modes (Table 4). These brain structures are involved in diverse neurophysiological and psychological functions, including sensory, locomotor, visceral, sleep, emotion, and cognition processes. The widely distributed network constituted by these brain regions may, therefore, be essential substrates for the broader therapeutic effects of acupuncture. This widespread and diverse modulation of brain contingent upon acupuncture is most likely related to the multiple central pathways for the compound signal inputs that are initiated through the biochemical and biophysical reactions of NAUs.

Despite methodological issues of nonspecificity discussed above, numerous studies have indicated that manual and electrical stimulation at real acupoints modulate a greater extent of brain areas and elicit more intense response compared to control points or non-acupoints in healthy volunteers [118–124]. Similar results have been observed for manual acupuncture versus acupressure [125], deep versus shallow electrical stimulation on the same acupoints [126], rotating versus nonrotating stimulation [120], and long versus short duration of manual acupuncture [127]. Moreover, acupuncture-induced needling sensation without sharp pain also modulates greater extent of brain areas compared to needling sensation with sharp pain [72, 128]. As acupoint-based NAUs contain relatively dense neural and neuroactive components with the predominance and concentration of somatosensory receptors and their afferent innervations, stimulation modes with greater depth, longer duration, and accompanying needling sensation would be expected to activate more neural and neuroactive components and wider spectrum of afferent fibers, producing stronger and longer-lasting NAU afferent signals. Therefore, more widespread and intense neuroimaging response appears to be a consequence of more efficient NAU stimulation.

### 8.2. Frequency-Specific Neurochemical Response to Electroacupuncture (EA)

It is well documented that many neurochemicals, in particular endogenous opiate peptides, 5-HT, and catecholamines, exhibit a frequency-dependent response in EA-produced analgesic effects [3, 129, 130].

Analgesic effects produced by EA at human and animal acupoints with muscle spindle-rich NAUs, such as Zu-San-Li (ST36) and San-Yin-Jiao (SP6), are closely associated with enhanced release of endogenous opiate peptides in CNS [130]. Moreover, low-frequency (2–15 Hz) EA and TENS exert antinociceptive effects by enhancing the release of central enkephalin, endomorphin, *β*-endorphin, and dynorphin that act at *μ*- and *δ*-receptors; whereas high frequency (100 Hz) produces antinociceptive effects by enhancing the release of dynorphin that mainly acts at *κ*-receptors [130]. Likewise, while both low- (4–10 Hz) and high- (100 Hz) frequency EA stimulation at Zu-San-Li (ST36) and Huan-Tiao (GB30) have been observed to produce a significant increase in immunoreactivity of 5-HT neuronal cells in the dorsal raphe nucleus and the nucleus raphe magnus, the high-frequency EA has more potent effects in increasing 5-HT activity in the rat dorsal raphe nucleus compared to low frequency [101, 102, 131, 132]. Both frequencies have similar effects in enhancing the activity of catecholaminergic neurons in the rat hypothalamus and the brainstem reticular formation [103]. Stimulation with only 6, 15, 21 Hz, but not 9, 12, 18, 24, 27, and 30 Hz, at Da-Ling (PC7) has been found to significantly increase the release of dopamine in the rat striatum [130]. These studies clearly indicate frequency-specific effects of EA in central neurochemical systems.

The frequency-specific neurochemical effects observed in acupuncture analgesia could be explained by different peripheral and central pathways transmitting NAU afferent signals produced by low- and high-frequency stimulation. It is well documented that low-frequency, high-intensity EA and TENS excite predominantly myelinated fibers (A*β* and A*δ*), whereas high-frequency EA mainly activates small-diameter myelinated A*δ* fibers and unmyelinated C fibers [133, 134]. Neuroimaging studies have further demonstrated differences in brain regions modulated and the nature of modulation between 2 Hz and 100 Hz frequency stimulation on Zu-San-Li (ST36) and San-Yin-Jiao (SP6), although both frequencies elicit some common brain regional activity in human subjects [135, 136]. The opioid gene expression pattern in the rat brain induced by 2 Hz is also different from 100 Hz [137]. These studies suggest that distinct central neurochemical response patterns are related to the differences in the predominant neural and neuroactive components of NAUs activated by low- and high-frequency stimulation. Based on the fact that high-frequency stimulation has more potent effects on 5-HT activity in the rat raphe nuclei [102, 131], it is likely that high-frequency-elicited NAU afferent impulses may be predominantly conveyed by the brainstem 5-HT neuronal system-relayed pathways.

### 8.3. The Normalizing Effects of Acupuncture in Pathological Conditions

Numerous clinical studies have revealed that acupuncture treatment is capable of reversing and even normalizing abnormal neuroimaging activity in patients with chronic pain [138–140], cerebral palsy [141], chronic stroke [142–145], Parkinson's disease [146], Alzheimer's disease [147], major depressive disorder [148, 149], and heroin addiction [150]. Moreover, most reversal and normalization of neuroimaging signals are correlated with clinical improvement.

Similar effects have been observed in normalizing neurochemical abnormalities in depressive conditions, showing that EA treatment protects against decreased 5-HT and catecholamine in depressed patients [151, 152] and in animal models of depression [153, 154]. EA combined with antidepressant drugs even potentiates antidepressant effects in depressed patients [155, 156].

Consistent with this, both acute and repeated EA normalizes behavioral and biochemical abnormalities in various stressed animal models, including immobilization [105, 157–163], maternal separation [164–166], chronic mild stress [167, 168], surgical trauma [169], chronic administration of corticosterone [160], cold stimulation [170], tooth-pulp stimulation [171], and mechanical colon distention [172].

Acupuncture therapy is also beneficial in treating visceral disorders, particularly functional gastrointestinal disorders [173, 174], heart rate variability [175], hypertension [176], urinary incontinence [177], and asthma [178]. It is well documented that the principal mechanism of visceral effects of acupuncture is to rearrange the balance of sympathetic and parasympathetic activity via somato-autonomic reflex [129, 179, 180]. NAU afferent signals are transmitted by multiple peripheral and central neural pathways to different levels in CNS, mainly the spinal cord, the brainstem, and the hypothalamus, where they are relayed to visceral organs via autonomic efferent fibers, neuroendocrine, and neuroimmune systems. This ultimately results in a rebalance of sympathetic and parasympathetic activity [129, 179, 180].

Taken together, normalizing neuroimaging, neurochemical and behavioral abnormalities in neuropsychiatric conditions, as well as rebalancing sympathetic and parasympathetic activities in visceral disorders, represent broad therapeutic effects of acupuncture at systemic and central levels. These effects are achieved initially through an NAU-based local mechanism. There is an extensively bidirectional communication between the brain and peripheral immune system [181]. For instance, peripheral inflammatory information can be transmitted through peripheral sensory nerves to the visceral function-regulated brain regions, such as the solitary nucleus and the hypothalamus [182]. Changes in peripheral immune functions have been implicated in the etiology and pathogenesis of many neuropsychiatric syndromes [183–185]. Numerous studies confirm that such neuropsychiatry-associated peripheral immune changes are reflected in the subtle imbalance of immune mediators [184, 186]. Pro- and anti-inflammatory factor imbalance in peripheral tissues has also been linked with various pain disorders [187–190], and imbalances of immune mediators have been widely observed in major depression [191, 192], anxiety disorders [191, 193], sleep disorders [194], and neuroendocrine disorders [195].

Acupuncture treatment has been shown to restore the balance between pro- and anti-inflammatory factors in depressed patients [192] and reestablish immunological balance in rats with experimental autoimmune encephalitis [196] and exposed to traumatic condition [197], while clinical symptoms and animal abnormal behavior were improved. It is, therefore, suggested that acupuncture stimulation improves central pathophysiology by rearranging the balance of peripheral neuroactive mediators and modulating NAU afferent signals. The ultimate result is the normalization of neuroimaging, neurochemical, and behavioral abnormalities and a rebalance of visceral autonomic activities. This explanation is consistent with the philosophy of TCM that an important mechanism of acupuncture effects is to rebuild the balance of *Yin* and *Yang*.

## 9. Conclusions

1. NAU is a hypothetical concept that represents the collection of local neural and neuroactive components distributed in the skin, muscle, and connective tissues activated by an acupuncture needle that is inserted into a designated point on the body, and mechanical or electrical stimulation is delivered. The traditionally defined acupoints could be defined as an anatomical landmark system that indicates local sites where NAUs may contain relatively dense and concentrated neural and neuroactive components, upon which acupuncture stimulation would elicit a more efficiently physiological and therapeutic response compared to non-acupoints.
2. Somatosensory receptors and their afferent fibers are the major neural components of NAUs and play the central role in the production of NAU afferent signals. Neuroactive components of NAUs are non-neuronal tissues and cells that release various mediators capable of modulating NAU afferent signals via local biochemical reactions. Biophysical reactions of NAUs are triggered by the activation of mechanoreceptors in NAUs due to mechanical pressure and tissue distortion induced during manual manipulation. NAU-based local mechanism plays an equally important role in acupuncture analgesia as central mechanisms.
3. Different types of NAUs are associated with different NAU afferent impulses and components of needling sensation. The biochemical and biophysical reactions of NAUs dominantly activate small-diameter (A*δ* and C) and myelinated afferent fibers (A*β* and A*δ*), respectively. The induction of aching, soreness, and warmth of needling sensation are closely associated with the activation of A*δ* and C fibers in NAUs, whereas numbness, heaviness, and distension are mainly related to the activation of A*β* and A*δ* fibers.
4. Multiple central neural pathways convey NAU afferent impulses. The spinothalamic and spinoreticular tracts dominantly transmit biochemical reaction-evoked signals, whereas the dorsal column-medial lemniscus tract mainly transmits mechanoreceptor-activated signals. The trigeminal sensory pathway that conveys NAU afferent signals from the trigeminal territory has closer connections with the brainstem reticular formation, particularly 5-HT and catecholaminergic neuronal systems, which play a pivotal role in the modulation of broad effects of acupuncture. DCEAS has superior effects in the treatment of neuropsychiatric disorders.
5. A distributed network of widespread brain regions that respond to acupuncture provides the neural substrate for the broad therapeutic effects of acupuncture. The more widespread and intense brain regional response may be a consequence of more efficient NAU stimulation. A frequency-specific neurochemical response in the CNS may be related to differential response of NAUs to low- and high-frequency EA stimulation and different peripheral and central pathways. Acupuncture has broad effects of normalizing neuroimaging, neurochemical, and behavioral abnormalities in neuropsychiatric disorders as well as regulating autonomic activities in visceral disorders. These effects may be achieved initially by rearranging the subtle balance of neuroactive mediators and modulating NAU afferent impulses.

## 10. Implications and Future Directions

The establishment of the conception of NAU and its differentiation from acupoint not only provide an alternative theoretical approach into acupuncture research, but also bring many implications and impacts on further directions.

1. The NAU-based local mechanism by which acupuncture stimulation locally modulates NAU biochemical reactions provides an important scientific rationale for traditional multiple-needling techniques, such as seven-star needling, plum-blossom-like needling, and round-needling, most of which are specifically used to treat focal lesions and pain conditions. The clarification of differences in local effects between multiple-needling and other needling techniques will help develop more efficient and specific acupuncture treatment regimens. Although the local roles of some NAU neuroactive mediators are well defined in acupuncture analgesia [15, 30, 31, 47, 48], most NAU mediators, as listed in Table 1, need to be further examined.
2. While most previous studies have placed the emphasis on the lateral funiculus of the spinal cord; the dorsal column-medial lemniscus tract and the trigeminal sensory pathway have received relatively less attention. As mechanoreceptor-activated signals dominate in most NAU afferent impulses, particularly in muscle-spindle-rich and tendon-organ-rich NAUs, the role of the dorsal column-medial lemniscus tract in acupuncture effects deserves to be further clarified. As an efficient stimulation mode, DCEAS was developed based on the neuroanatomical rationale that NAUs in the trigeminal territory have intimate connections with the brainstem reticular formation. Neurophysiological and neuroimaging studies of this novel acupuncture mode will provide direct evidence to prove its efficiency.
3. In clinical practice, acupuncture treatment regimens generally consist of multiacupoints located in different parts of the body. Empirical and experimental evidence suggests that the combination of local and distant acupoints produces greater treatment effects than the sum of single acupoints. Different central mechanisms are implicated in processing acupuncture signals from acupoints located in homeo- and heterosegmental spinal nerve territory [83]. Simultaneous stimulation of different acupoints appears to elicit more widespread and intense brain regional response [198]. Given that superior therapeutic response is associated with synergistic or additive effects of NAUs at local and systemic levels, the clarification of this relationship will provide valuable information in the development of more efficient acupuncture treatment regimens.
4. Sham acupuncture often serves as a control in basic and clinical acupuncture research. The two most commonly used sham procedures are (i) insertion of acupuncture needles into control points generally defined at a certain distances (usually 1–3 cm) from acupoints and (ii) noninserted placebo needling on the same acupoints [199]. These control procedures were initially designed to differentiate specific acupuncture effects at acupoints from non-acupoints. Nevertheless, as mentioned earlier, the most notable difference between most acupoints and non-acupoints is the relatively higher density of certain neural and neuroactive components with predominance and concentration of somatosensory receptors and their afferent fibers in acupoint-based NAUs. Clinically, it might be difficult to differentiate the effects of acupoints from adjacent points; even if needles are not inserted into the skin at non-acupoints, it may excite mechanoreceptors of NAUs. This could, at least in part, explain why most clinical studies have failed to demonstrate superior efficacy in “real” (or called “true”, verum or genuine) acupuncture treatment regimens compared to sham or placebo regimens; sham acupuncture intervention even displays superior efficacy compared to inert placebo acupuncture [1, 200]. In order to identify the systemic effects of acupuncture, a valid control design should completely block the production of NAU afferent impulses. For this purpose, the utilization of modified needles with local anesthetic drugs might be considered.
5. Inadequate “dose” is thought to be an important factor in the failure of many clinical studies of acupuncture to achieve positive treatment outcomes [76]. Indeed, our recent meta-analysis of acupuncture therapy in depressive disorders [201] and a systematic review [202] have confirmed that most clinical trials did not include criteria for either qualitative or quantitative adequacy of acupuncture treatment regimens. Acupuncture “dosage” in fact represents both local and systemic efficiency of NAU stimulation. Local efficiency can be reflected in changes in local NAU-associated biochemical and electrodermal indices; systemic efficiency may be indicated in the needling sensation, neuroimaging, or neurochemical response recorded in CNS. While the verbal report of the intensity of needling sensation as a subjective scale has been demonstrated to be a valid psychological indicator for the intensity of acupuncture stimulation [203], the exploration of NAU-associated neurophysiological and neurochemical indicators may result in the discovery of objective measures of acupuncture “dosage.”

## Figures and Tables

**Table 1:** Major non-neuronal neuroactive mediators involved in the modulation of NAU afferent fiber excitability.

Mediators	Non-neuronal cells releasing neuroactive mediators	Receptors and actions on NAU afferent fiber terminals	Effects on NAU afferent fiber excitability	Reference
Serotonin (5-HT)	Platelets, mast cells	5-HT_3_ receptor	+	[17]
		5-HT_1_ receptor	−−	[203]
Noradrenaline (NA)	Mainly released from sympathetic nerve varicosities. Epidermal cells may be NA-storing cells.	*α*_2_ Receptors	−−	[51]
Acetylcholine	Keratinocytes and injured efferent fiber terminals	Muscarinic M_2_ receptor	−−	[204]
Histamine	Mast cells	H_3_ receptor	−−	[17]
		H_1_ receptor	+	
Glutamate/aspartate	All skin epithelial cells and macrophage	Autoreceptors (?)	−− (?)	[35, 36, 205]
*γ*-aminobutyric acid (GABA)	Macrophages and lymphocytes	GABA_A_ receptors	−−	[206, 207]
*β*-endorphin	Keratinocytes, melanocytes, dermal fibroblasts, and leukocytes	*μ*-opiate receptors	−−	[47, 48, 208]
Substance P (SP)	Mast cells, fibroblasts, platelets, keratinocytes, and macrophages.	Autoreceptor (?)	−− (?)	[14, 37, 38, 46]
Calcitonin gene-related peptide (CGRP)	Epithelial cells, T cells, macrophages	Autoreceptor (?)	−− (?)	[39, 209, 210]
Somatostatin (SS)	Merkel cells, keratinocytes	SS receptors	−−	[21, 211–213]
Nitric Oxide (NO)	Local tissues	Inhibits SP release from primary afferent terminals and enhances acetylcholine and *β*-endorphin.	−−	[50, 214, 215]
ATP/cGMP	Epidermal cells	P2X and P2Y receptor	−−	[31, 214, 216]
Adenosine	Degraded from ATP released in response to mechanical, electrical, or heat stimulation.	A_1_ receptor	−−	[31]
Bradykinin	Local tissues and cells	B_1/2_ receptors	+	[217–219]
Cytokines (IL-1*β*, IL-6, IL-8, and TNF-*α*)	Local tissues and cells	Stimulate afferent fibers and augment their excitability	+	[217–221]
Cytokines (IL-4 and IL-10)	Local tissues and cells	Inhibits the production of inflammatory pain signals in afferent terminals	−−	[220, 222]
Prostaglandins	Local tissues and cells	EP receptors	+	[221, 223]

^a^Question marks (?) indicate to be determined.

**Table 2:** Classification of NAUs based on a predominance of somatosensory receptors.

Type	Definition and characteristics	Related acupoints
Muscle-spindle-rich NAUs	When an acupuncture needle is inserted, a large portion of the inserted needle body is surrounded by muscle fibers. Muscle spindles are the major neural components in this type of NAUs. There are about 210–2,860 muscle spindles/cm^3^ in muscle tissues in the back of the Bladder-Meridian [225].	Nearly 60% acupoints located on thick muscle areas contain this type of NAUs, for example, Zu-San-Li (ST36), He-Gu (LI4), and Huan-Tiao (GB30). Most acupoints can be performed with large-scale manual techniques.

Cutaneous-receptor-rich NAUs	Relatively dense and concentrated cutaneous receptors dominate in NAUs. About 100–240 encapsulated cutaneous receptors/mm^2^ and 300 free nerve endings/mm^2^ are distributed in the cutaneous tissues of the finger pads [226].	Most acupoints containing this type of NAUs are located on the finger pads, palms, plantar areas, and the surrounding of the lips, for example, Shao-Shang (LU11), Lao-Gong (PC12), and Ren-Zhong (GV26). Only prick and shallow needling can generally be performed on these acupoints.

Tendon-organ-rich NAUs	Tendon organs, Ruffini and Pacinian corpuscles dominate in NAUs.	Most acupoints containing this type of NAUs are located around the elbow, wrist, knee, and ankle joints, for example, Chi-Ze (LU5), Da-Ling (PC7), Du-Bi (ST35), and Jie-Xi (ST41).

**Table 3:** The putative relationship between NAU properties and needling sensation.

NAU properties	Components of needling sensation	
	Aching/soreness/warmth	Numbness/heaviness/distension
Type of NAUs	Cutaneous-receptor-rich NAUs with a predominance of nociceptors.	Muscle-spindle- and tendon-organ-rich NAUs.
NAU reactions	Biochemical reaction	Biophysical reaction
Dominant afferent fibers	A*δ* and C	A*β* and A*δ*
Acupuncture stimulation modes^a^	Prick; shallow needling; high-frequency EA; laser acupuncture; heat acupuncture.	Most manual techniques in gentle and repetitive manipulation; low-frequency, high-intensity EA and TENS.

^a^Low-frequency, high-intensity EA and TENS is thought to dominantly activates myelinated fibers (A*β* and A*δ*), whereas high-frequency EA mainly activate small-diameter myelinated A*δ* fibers and unmyelinated C fibers [133, 134].

^b^EA, electroacupuncture; TENS, transcutaneous electrical nerve stimulation.

**Table 4:** Brain regions with common neuroimaging response to acupuncture stimulation and acupuncture-associated neurophysiological and psychological effects^a^.

Brain regions	Functional neuroimaging response to acupuncture	Acupuncture-associated effects
Primary somatosensory cortex	Activation	Pain and mechanoreceptor-activated signals
Prefrontal cortex	Activation	Cognition and emotion
Insula	Activation/deactivation	Pain
Anterior cingulate cortex (ACC)	Deactivation	Pain, attention, memory, and emotion
Hypothalamus	Activation	Autonomic, neuroendocrine, visceral function, and stress-processed center
Amygdala/hippocampus	Activation	Encoding emotional signals and short-term memory
Thalamus	Activation	Pivotal relay station processing sensory inputs
Cerebellum	Activation	Locomotor coordination, higher-order cognitive and emotional function
Periaqueductal grey (PAG) and raphe nuclei	Activation	Modulating opioidergic and serotonergic activity, involved in pain, sleep, and consciousness.

^a^Some contents were extracted from [112].
